# Functionally Structured Genomes in *Lactobacillus kunkeei* Colonizing the Honey Crop and Food Products of Honeybees and Stingless Bees

**DOI:** 10.1093/gbe/evv079

**Published:** 2015-05-06

**Authors:** Daniel Tamarit, Kirsten M. Ellegaard, Johan Wikander, Tobias Olofsson, Alejandra Vásquez, Siv G.E. Andersson

**Affiliations:** ^1^Department of Molecular Evolution, Cell and Molecular Biology, Science for Life Laboratory, Uppsala University, Sweden; ^2^Medical Microbiology, Department of Laboratory Medicine, Lund University, Sweden

**Keywords:** genome organization, *Lactobacillus kunkeei*, honeybee, genome reduction, recombination

## Abstract

*Lactobacillus kunkeei* is the most abundant bacterial species in the honey crop and food products of honeybees. The 16 S rRNA genes of strains isolated from different bee species are nearly identical in sequence and therefore inadequate as markers for studies of coevolutionary patterns. Here, we have compared the 1.5 Mb genomes of ten *L. kunkeei* strains isolated from all recognized *Apis* species and another two strains from Meliponini species. A gene flux analysis, including previously sequenced *Lactobacillus* species as outgroups, indicated the influence of reductive evolution. The genome architecture is unique in that vertically inherited core genes are located near the terminus of replication, whereas genes for secreted proteins and putative host-adaptive traits are located near the origin of replication. We suggest that these features have resulted from a genome-wide loss of genes, with integrations of novel genes mostly occurring in regions flanking the origin of replication. The phylogenetic analyses showed that the bacterial topology was incongruent with the host topology, and that strains of the same microcluster have recombined frequently across the host species barriers, arguing against codiversification. Multiple genotypes were recovered in the individual hosts and transfers of mobile elements could be demonstrated for strains isolated from the same host species. Unlike other bacteria with small genomes, short generation times and multiple rRNA operons suggest that *L. kunkeei* evolves under selection for rapid growth in its natural growth habitat. The results provide an extended framework for reductive genome evolution and functional genome organization in bacteria.

## Introduction

Symbiotic associations between bacteria and insects are common in nature. These include mutualistic bacteria that provide beneficial functions, such as supplementing the host diet with nutritional compounds (Moran et al. 2008; Moya et al. 2008; Toft and Andersson 2010). Increasingly investigated are extracellular bacteria of the gut, for which host beneficial functions are less obvious. Metagenomic studies have been applied to the insect gut microbiota to examine how taxonomic compositions and metabolic functions correlate with geography, seasons, and other environmental factors (Engel and Moran 2013). However, despite the recent progress in cataloging the bacterial species in the guts of insects, we know very little about the ecological role that the individual members play.

The gut microbiota of the honeybee *Apis mellifera* has gained particular attention due to the recent losses in managed honeybee colonies and the importance of honeybees as pollinators of diverse agricultural crops (Evans and Schwarz 2011). Cultivation and 16 S rRNA profiling experiments have shown that the mid- and hindgut consist of only eight bacterial phylotypes (Martinson et al. 2011; Moran et al. 2012; Sabree et al. 2012), including *Lactobacillus* and *Bifidobacterium* spp. (Olofsson and Vasquez 2008; Vásquez and Olofsson 2009; Vásquez et al. 2009; Olofsson et al. 2011; Vasquez et al. 2012). Genome sequencing of Lactobacillus and Bifidobacteria isolated from the gut of *A. mellifera* has revealed extensive gene content variation despite low sequence divergence levels at the 16 S rRNA genes (Ellegaard et al. 2015). Almost 50% of the accessory genes were estimated to code for proteins involved in carbohydrate metabolism and transport functions, consistent with adaptation to an environment with a rich and variable content of carbohydrates.

In the foregut (honey crop), the dominant species is *Lactobacillus kunkeei*, as inferred from both cultivation experiments and 16 S rRNA surveys (Olofsson and Vasquez 2008; Vasquez et al. 2012; Anderson et al. 2013; Vojvodic et al. 2013). A sampling of 750 bacterial isolates from the honey crop of nine *Apis* species and three stingless bee species from Asia, South America, and Africa identified 44% as *L. kunkeei* (Vasquez et al. 2012). This suggests that *L. kunkeei* is globally present in honeybees and stingless bees.

The type strain of *L. kunkeei* was isolated from a spoiled wine fermentation (Edwards et al. 1998), and phenotypic tests showed that *L. kunkeei* is an obligate fructophile that prefers fructose as a source of sugars, and is able to grow well on glucose only in the presence of fructose and external electron acceptors, such as oxygen or pyruvate (Endo et al. 2012; Neveling et al. 2012). Therefore, it is likely to be viable in any habitat with these characteristics. Indeed, *L. kunkeei* is not only present in the honey crop but also abundant in beebread (Vásquez and Olofsson 2009; Anderson et al. 2013), as well as in pollen and bee larvae (Vásquez and Olofsson 2009; Vojvodic et al. 2013). Additionally, it has been cultivated from flowers and fruits using highly selective culturing methods (Endo et al. 2012; Neveling et al. 2012; Anderson et al. 2013). However, 16 S rRNA screening of the nectar from bee-pollinated flowers of three plant species showed that more than 83% of the sequences were from Proteobacteria and none from *Lactobacillus* (Fridman et al. 2012). Furthermore, using this methodology, *L. kunkeei* was not detected in the nectar of flowers but it was identified on the surface of honeybees visiting these same flowers (Aizenberg-Gershtein et al. 2013). Thus, it has not been established whether flowers represent one of its growth niches, or whether *L. kunkeei* is only deposited in flowers through bee pollination.

The ecological role of *L. kunkeei* within bees and beehives is also unknown. In vitro studies have shown that *L. kunkeei* isolated from honeybees can inhibit the growth of bacteria and yeast, including pathogens of bees and humans (Forsgren et al. 2010; Vasquez et al. 2012; Butler et al. 2014; Olofsson, Butler, et al. 2014). Additionally, it was demonstrated that the morbidity of honeybee larvae by bee pathogens was reduced if the diet was supplemented with a “cocktail” of *L. kunkeei* and other lactic acid producing bacteria (Forsgren et al. 2010; Vasquez et al. 2012). Finally, it has been shown that *L. kunkeei* produces extracellular proteins during cultivation in the laboratory when lipopolysaccharides and other stress-inducing compounds are added to the growth medium (Butler et al. 2013). Based on these results it was hypothesized that *L. kunkeei* plays a role in bee health by inhibiting the growth of bacteria and fungi that are harmful to the bees (Forsgren et al. 2010; Vasquez et al. 2012).

A key question is whether *L. kunkeei* has codiversified with the bees, or whether it can jump between bee species. With the exception of *Lactobacillus apinorum* Fhon13*,* which was recently classified as a separate species (Olofsson, Alsterfjord, et al. 2014), the 16 S rRNA sequences of the isolated *L. kunkeei* strains differ by at the most one single nucleotide and are therefore inadequate as molecular markers to demonstrate codiversification, or the lack thereof. To test the hypothesis that bacteria and bees have codiversified, we have sequenced and compared the genomes of *L. kunkeei* Fhon2 and *L. apinorum* Fhon13 isolated from *A. mellifera* with ten additional strains isolated from bees from the Apini and Meliponini tribes. We have also searched for putative host-adaptive traits and examined frequencies of recombination and horizontal gene transfers for strains isolated from different host species.

## Materials and Methods

### Experimental Methods

#### Genome Sequencing

The bacterial strains were isolated from honey crops, bee bread, corbicular bee pollen, and honey originating from the number of locations depicted in table 1 as previously described (Vasquez et al. 2012). Briefly, crops were analyzed from 10 to 20 bees from each bee species. Honey (1 g) and bee bread (1 g) were collected directly from the colonies. Corbicular bee pollen was pulled off from the legs of approximately ten incoming foragers. The samples were then mashed, vortexed, and diluted. Pure isolates were obtained by cultivation during 3 days on supplemented de Man, Rogosa, and Sharpe (MRS) plates (Oxoid) (2% fructose and 0.1% l-Cysteine) at 35**°**C during anaerobic conditions. Strains *L. kunkeei* Fhon2 and *L. apinorum* Fhon13 were isolated using the same procedure from the crop of *A. mellifera mellifera* from an apiary in Northern Sweden.

**Table 1:** Sampling Location, Host, Original Names and Suggested Abbreviations for the Strains of the *Lactobacillus kunkeei* Species Complex

Sampling Location	Host	Original Strain Name	Name Used in This Study
Borneo (Malaysia)	*Apis andreiformis*	AnhonRo7	LAan
Borneo (Malaysia)	*Apis cerana*	CepoRo6	LAce
Borneo (Malaysia)	*Apis dorsata*	DohmRo1	LAdo
Thailand	*Apis florea*	FLHSR7	LAfl
Borneo (Malaysia)	*Apis koschevnikovi*	Kobbto5	LAko
Nepal	*Apis laboriosa*	La1honRo9	LAla
Sweden	*Apis mellifera*	Fhon2N	Fhon2
Sweden	*Apis mellifera*	Fhon13N	Fhon13
Indonesia	*Apis nigrocincta*	Nigbbto6	LAni
Borneo (Malaysia)	*Apis nuluensis*	nuhmRo20	LAnu
Mexico	*Melipona beecheii*	kemebb2to13	LMbe
Kenya	*Meliponula bocandei*	Mb2bbto8	LMbo
United States	Wine spoilage strain	YH-15	LK

DNA extracted from the strains *L. kunkeei* Fhon2 and *L. apinorum* Fhon13 isolated from *A. mellifera* was sequenced from a 6-kb paired-end library on a 454 FLX Roche instrument using Titanium chemistry, and from a paired-end Illumina library on a Miseq instrument (2 × 150 bp). All other *L. kunkeei* strains were sequenced from paired-end (2 × 150 bp) libraries on a Miseq instrument and single-end (100 bp) libraries on an Illumina HiSeq 2000 instrument, using standard Illumina protocols with chemistry v3.0. The sequencing was performed by MWG Eurofins Operon (Ebensburg, Germany).

#### Growth Curves

*Lactobacillus kunkeei* Fhon2 and *L. apinorum* Fhon13 were grown on a pollen medium (15% collected bee-pollen in water, sterilized at 120°C for 20 min). Bacterial strains were incubated at 35°C in an anaerobic condition. Absorbance was measured every 30 min at an OD_620_ without opening the tubes. The generation time was calculated in R (R development core team 2011) by fitting the absorbance measurements that belonged to the exponential phase to a linear model, and then dividing log(2) by the obtained coefficient.

#### Prevalence of *L. kunkeei* Fhon2 and *L. apinorum* Fhon13

Samples were taken from 27 beehives from two apiaries in Helsingborg, Sweden during the autumn (2 hives), winter (6 hives), spring (6 hives), and summer (13 hives). From each hive, samples were taken from ten honeybees (honey crop), 1 g honey, 1 g corbicular bee-pollen, and 1 g beebread. The samples were then dissolved and diluted in sterile PBS (Phosphate Buffer Saline). Pure isolates were obtained by growth on supplemented MRS plates under anaerobic conditions for 3 days at 35**°**C. Identification of the isolates was performed by polymerase chain reaction (PCR) amplification, sequencing, and plylogenetic analyses of the 16 S rDNA gene as previously described (Olofsson and Vasquez 2008; Vásquez and Olofsson 2009; Vasquez et al. 2012). The prevalence was measured as viable counts in cultivation on supplemented MRS (2% fructose and 0.1% l-Cysteine).

#### PCR Analyses

Bacterial DNA was isolated using the QIAamp DNA minikit (Qiagen, The Netherlands). The PCR reactions contained PCR buffer (75 mM Tris–HCl pH 8.8, 20 mM (NH_4_)_2_SO_4_, 0.1% (v/v) Tween 20), 1.0 U *Taq* DNA polymerase (Thermo Scientific, MA), 0.2 mM dNTPs (Thermo Scientific), and 0.2 μM of the primers. The annealing temperature was 46–48°C for 30 s. The PCR amplicons were sequenced with Sanger technology. The primers used for the PCR reactions were:

RecGFw (5′-TCTGGAATCAAGATTTTATCTTCGGT-3′),
RecGRev (5′-GTGCTTTCCCCATGATATCACC-3′),
LepA1Fw (5′-GCAGTTGAGCTAAAGTACCATTCTAAG-3′),
LepA2Rev (5′-ACTTCGTATTCACTACCACTGTTC-3′),
LepA2Fw (5′-CATGGATGTTGTGCAAGAACG-3′), and
LepA2Rev (5′-CTTCATCAGTTTGTAGAACAGCC-3)

### Genome Assembly and Annotation

#### Assembly and Annotation

The paired-end Illumina sequences were trimmed with Trimmomatic (Bolger et al. 2014), whereas the single-end sequences were cleaned by the sequencing provider. The genomes from strain Fhon2 and Fhon13 were assembled with Newbler (454 Life Sciences Corp., Roche, Branford, CR) using both 454 and Illumina data simultaneously. The genomes for which only Illumina data were obtained were assembled with Velvet (Zerbino and Birney 2008). Several k-mer sizes were explored, before choosing the final k-mer values between 89 and 95. The assemblies were further verified by mapping the Illumina reads onto the assembly with bwa (Li and Durbin 2009), calculating the coverage with samtools (Li et al. 2009), and plotting the result with R. The mapping was also manually inspected with Artemis (Rutherford et al. 2000) and synteny was evaluated by ordering the scaffolds with Mauve (Darling et al. 2010) and examining the result with Artemis Comparison Tool (Carver et al. 2005). Due to the detection of putative misassemblies in Velvet scaffolds, the contigs were extracted from all scaffolds and reordered to maximize synteny according to the assemblies of strains Fhon2 and Fhon13 (for which 454 paired-end data had been employed). Finally, the assemblies were arranged so that the beginning of the *dnaA* gene would define the start of the genomes.

The contigs of all obtained assemblies were concatenated and annotated by running the DIYA pipeline (Stewart et al. 2009) including the software Prodigal (Hyatt et al. 2010), tRNAscan (Lowe and Eddy 1997), RNAmmer (Lagesen et al. 2007), and genePRIMP (Pati et al. 2010). All coding sequence (CDS) features were used for Basic Local Alignment Search Tool (BLAST) comparisons (Altschul et al. 1990) against the uniprot database (UniProt Consortium 2014), and Fasta comparisons (Pearson 1990) against a local database made from all the proteomes from complete *Lactobacillus* genomes. Furthermore, hmm search as implemented by pfam_search.pl against the Pfam database (Punta et al. 2012) was used for domain identification. All genes were annotated as “partial” if they spanned a contig or scaffold border, and were accordingly manually shortened or split. All genes flagged by Geneprimp were manually inspected and called as pseudogenes if they contained frameshifts or truncations in comparison to their best BLAST hits. BLAST against the COG database (Tatusov et al. 2000) was performed for all CDSs, and were assigned whenever the two best BLAST hits belonged to the same COG and the comparison *E* value was lower than 0.01. The accession numbers for each one of the genomes are shown in supplementary table S1, Supplementary Material online.

#### Plasmid and Prophage Detection

Four criteria representing plasmid properties were used to examine a possible extrachromosomal nature for every contig: 1) Visually relevant changes in coverage, 2) presence of read pairs connecting the two extremes of the contig (i.e., indicating circularity), 3) higher similarity to plasmids than chromosomes when searches was performed using BLASTn or tBLASTx against the nt database, and 4) presence of genes with putative plasmid origin. Prophages were detected with PHAST (Zhou et al. 2011) and Prophage Finder (Bose and Barber 2006). Prophage Finder was first tested with default parameters and second with a more strict *E* value threshold (1e-3). PHAST served as confirmation for some of the regions detected by the stricter version of Prophage Finder. The gene content of each one of the predictions was assessed to discard false positives and the regions were compared between strains to establish homology relationships.

#### CRISPR Detection and Analysis

Putative CRISPR regions were detected by using the tool CRISPRfinder (Grissa et al. 2007). Their classification was done by identification of the associated *cas* genes and their order following the classification proposed by Makarova et al. (2011). The analysis was further confirmed by aligning the detected *cas1* gene sequences with the data sets analyzed by Makarova et al. (2011) and Horvath et al. (2009), trimming the alignments with trimAl (Capella-Gutierrez et al. 2009), and reconstructing their phylogeny with RAxML (Stamatakis 2014). The detected spacers were compared through BLASTn against the nt database at National Center for Biotechnology Information (NCBI) (Pruitt et al. 2005), against the presently studied genomes, and against a local database containing only plasmid and phage sequences from the NCBI database.

### Evolutionary Analyses

#### Phylogenetic Analysis

Selected 16 S rRNA sequences from *Lactobacillus* strains isolated from insects were retrieved from public repositories. Additionally, the SILVA database (Quast et al. 2013) was surveyed for SSU rRNA sequences that were at least 60% identical to the predicted 16 S rRNA sequence from Fhon2. The resulting sequences were aligned with SINA (Pruesse et al. 2012) against the curated SILVA alignment for SSU sequences named Ref NR 99 (Quast et al. 2013), and the resulting alignment was then trimmed with trimAl for all gaps present in more than 50% of the sequences. The pruned alignment was used for phylogenetic reconstruction with RAxML using the GTRGAMMA model and 100 bootstrap pseudoreplicates.

To infer a bacterial genome phylogeny, OrthoMCL was run using the genomes for *L. kunkeei* and *Lactobacillus sanfranciscensis.* The recommended inflation value (1.5) plus a very stringent value (5) was selected to study the robustness of the detection. The 790 obtained single-copy clusters were confirmed to be identical in the two analyses. These were individually aligned at the protein level with mafft-linsi, back-translated into nucleotides with TranslatorX (Abascal et al. 2010), trimmed with trimAl for sites with over 50% gaps, and concatenated. RAxML was used with the GTRGAMMA model and 1,000 bootstraps to infer a core genome phylogeny. Confirmation of the obtained results was done by performing two runs of MrBayes (Ronquist and Huelsenbeck 2003) using the GTR (general time reversible) substitution model until convergence after 65,000 generations.

The host phylogeny was taken from the literature (Arias and Sheppard 2005; Raffiudin and Crozier 2007; Lo et al. 2010) from which a well-supported consensus tree was manually reconstructed.

Sequences obtained by PCR were aligned with Mafft and trimmed with TrimAl of all sites with greater than 50% gaps. Finally, all sequences that contained less than 50% of the length of the alignment were excluded. Phylogenetic trees were reconstructed with RAxML with the GTRCAT, and visualized with FigTree. The identity of supported clades was confirmed using MrBayes as explained above, for 80 million (*lepA* amplicons) and 20 million (*recG*) generations.

#### Ancestral Reconstruction

Complete genomes for all species within the Lactobacillaceae and Leuconostocaceae families were retrieved (if several genomes for a species were present, only the one from the first alphabetically ordered strain was kept), and three additional sequences were chosen to serve as outgroup: *Enterococcus faecalis*, *Lactococcus lactis*,** and *Streptococcus pyogenes.* Ortholog detection was done with OrthoMCL (Li et al. 2003) for the whole set of 50 genomes, using the recommended inflation value (1.5). All clusters containing only *L. kunkeei* genes were assigned to be unique to this clade.

A core genome phylogeny for a subgroup of these genomes was inferred based on 530 single-copy orthologs present in all *L. kunkeei* species complex genomes plus *Lactobacillus buchneri*, *Lactobacillus brevis*, *Lactobacillus plantarum*, *Lactobacillus mesenteroides*, *Leuconostoc gelidum*,** and *Leuconostoc kimchii*, the latter three serving as outgroups, using RAxML with the PROTCATLG model. The topology of the resulting tree excluding the outgroups was used to map all changes in ortholog groups defined by OrthoMCL. Gains and losses for each cluster were obtained using generalized parsimony with ACCTRAN in PAUP* 4.0b10 (Wilgenbusch and Swofford 2003) using the following costs for events: 10 for ortholog acquisition, 5 for ortholog loss, 1 for the first gene duplication, and 0.2 per copy for all other copy-number variations.

#### Functional Organization Assessment

For each genome, the distance of each gene to the origin of replication was calculated and the genes were classified by similarity to different functional COG categories. A Kruskal–Wallis test was performed to assess whether the COG categories had significantly different genome distances to the origin of replication. As a post hoc analysis, every pair of categories was compared with a Mann–Whitney test and performed with a Bonferroni correction. Circular genome plots were made with DNAPlotter (Carver et al. 2009), and comparative genome plots were done with the package GenoPlotR (Guy et al. 2010).

#### Giant Gene Analysis

The large gene region in every genome was analyzed with Artemis. Each of the orthologous clusters detected by OrthoMCL was aligned with the Mafft, Muscle, and ProbCons algorithms, and a consensus was built with M-Coffee (Wallace et al. 2006). Their orthology relationships were assessed by using the MCL algorithm (Enright et al. 2002) and further inspection of within-group multiple alignments and pairwise Smith–Waterman alignments as implemented by EMBOSS (Rice et al. 2000). Every gene was blasted against the nr database from NCBI and every orthologous group alignment was compared with the same database by Psi-BLAST (Altschul et al. 1997). Their domain structure was analyzed by comparison through InterProScan (Zdobnov and Apweiler 2001) and additional local hmm searches against the Pfam (Finn et al. 2014) and SCOP (Andreeva et al. 2008) databases. Finally, homology-based structural analyses were performed for the proteins of Fhon2 using Phyre2 (Soding 2005) over fragments of 2,000 amino acids or less.

#### Recombination Analyses

Individual phylogenies were inferred for each of the 790 single-copy orthologs of the *L. kunkeei–L. sanfranciscensis* data set with RAxML by constructing 100 rapid bootstrapped trees and a single slow best tree using the GTRCAT approximation. The trees were explored with Newick utilities (Junier and Zdobnov 2010) and custom perl scripts. The conflicting tree topologies for the A phylogroup were assessed by performing the one-sided KH, SH, and ELW tests with Tree-puzzle (Schmidt et al. 2002) with the Tamura–Nei model on six variations of the main *L. kunkeei* topology: The three possible arrangements of the A phylogroup plus three control alternatives: A switch from the main topology for the placement of Fhon2 and LMbo, Fhon2 and LAan, and Fhon2 and LAko. The site-likelihoods obtained from Tree-puzzle were fed to Consel (Shimodaira and Hasegawa 2001) in order to perform the AU test. d*S* values were calculated using the Yang–Nielsen method with the yn00 program, included in paml 4.5 (Yang 2007).

To estimate the overall ratio at which recombination and mutation events (r/m) had generated substitutions, ClonalFrame (Didelot and Falush 2007) was run on the nucleotide sequences of 25 housekeeping genes: *addA, adk, coaA, dnaA, dnaK, gyrA, gyrB, ileS, lepA, leuS, ligA, mnmE, mnmG, pheS, recA, recF, recG, rexB, rplB, rpoA, rpoB, rpoC, rpsC* and *truA,* for 20,000 generations and 100 generations between measures, and checked for convergence. Additionally, the Phipack package (Bruen et al. 2006) was used to evaluate recombination over the alignments of the 790 single-copy ortholog clusters for *L. kunkeei–L. sanfranciscensis* data set. Only the genes for which the three analyses included in the package (NSS, Maxchi, and Phi) yielded a *P* value below 0.01 were taken as positive for recombination.

## Results

### Sequencing the *L. kunkeei* Genomes

#### Strains, Hosts, and Seasonal Variations

We selected 12 bacterial strains for whole-genome sequencing from a previously published sampling of 750 lactic acid producing bacteria isolated from the honey crop as well as from honey, beebread, or pollen of honeybees (Vasquez et al. 2012). Two strains, *L. kunkeei* Fhon2 and *L. apinorum* Fhon13, were from *A. mellifera mellifera*, and one *L. kunkeei* strain was isolated from each of the other eight recognized *Apis* species and from two species of the *Meliponini* tribe (table 1). The seasonal abundance of *L. kunkeei* and *L. apinorum* in *A. mellifera* was investigated during four consecutive seasons by sampling beehives from the same apiary. These cultivation experiments showed that *L. kunkeei* was highly abundant in the honey crop, honey, beebread, and pollen during spring and summer, while being nearly absent during fall and winter (supplementary fig. S1, Supplementary Material online).

#### Sequencing, Assembly, and Annotation

Genomic DNA was extracted from each of the selected strains after cultivation in the laboratory. The strains grew rapidly under in vitro growth conditions, with an estimated generation time of 55 min for Fhon2 and Fhon13 in media that included 15% sterilized bee pollen (supplementary fig. S2, Supplementary Material online). Genome sequence data were collected from all 12 strains as well as from the type strain of *L. kunkeei* YH-15 ATCC 700308 (LK). The sequence data from Fhon2 and Fhon13 were assembled into single scaffolds that contained 49 and 23 contigs, which covered more than 99% of the assembly, plus 6 and 10 small contigs, respectively (supplementary table S2, Supplementary Material online). The assembled contigs of the other genomes were ordered with the aid of the single scaffolds for Fhon2 and Fhon13.

In each genome, the GC-skew curves displayed the characteristic shifts at the two opposite positions in the genomes (supplementary fig. S3, Supplementary Material online), providing indirect evidence that the contigs of all strains were ordered correctly. The intergenic region between *rpmH* and *dnaA* in the gene string *yidC-rnpA-rpmH-dnaA-dnaN-recF-gyrAB* coincided with one of the shifts in GC-skew values and also with a change of the strand that contained most of the genes, and was consequently annotated as the origin of replication (*ori*). The opposite GC-skew shift coincided with another switch in the strand gene content asymmetry and the presence of a consensus sequence for *dif* sites, identified previously at the terminus of replication in Firmicutes, Actinobacteria, and γ-Proteobacteria (Hendrickson and Lawrence 2007).

The genome sizes ranged from 1.42 to 1.59 Mb with a genomic G + C content of 35–38% (supplementary table S3, Supplementary Material online). They were predicted to contain 1,268–1,364 protein-coding genes, 45–65 tRNA genes, and 3–4 rRNA operons. A prophage of about 40 kb was detected in Fhon13, LAfl and Fhon2, and similar sequences were detected in single contigs in LMbe and LMbo. The latter two sequences were of higher coverage than the genomes overall and assembled into single, circularly permutated contigs that contained read pairs that mapped to the two ends, suggesting that they represent an actively replicating phage. Short contigs of 7.5–8.7 kb, likewise putatively coding for a prophage, were identified in LMbe, LAnu, and LAdo. Two additional unique prophages of 11.3 and 22.3 kb were identified in LK and LAan, respectively. Finally, three plasmids of 5.6–26 kb were identified in LAce, LAan, and LAfl.

### Phylogenetic Relationships

**Fig. 1.—:**
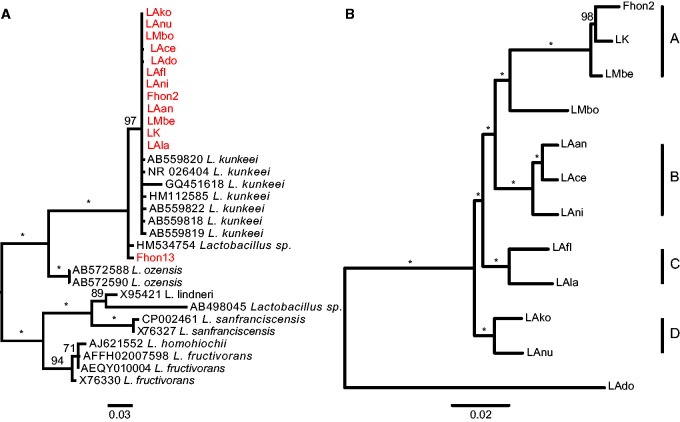
Phylogenetic relationships of the *L. kunkeei* species complex. The phylogenetic trees were inferred from (*a*) 16 S rRNA sequences and (*b*) a concatenated nucleotide alignment of 790 genes single-copy orthologs in the *L. kunkeei* species complex and *L. sanfranciscensis.* For ease of visualization, only the subtrees including the *L. kunkeei* clade are shown. Nodes with bootstrap support values of 100% are indicated with asterisks. The ancestral node in (*a*) was supported by 92% bootstrap support. The trees were inferred with the maximum-likelihood method. The same topology of the tree presented in (*b*) and similar branch lengths were obtained by Bayesian analysis, in which all nodes had a posterior probability of 1. Abbreviations of strain names are defined in table 1.

A comparison of a 1,408-bp-long alignment of the 16 S rRNA gene showed that eight of the isolated strains were identical to the type strain, *L. kunkeei* YH15, whereas strain LAdo contained a single polymorphism, and strains LAko and LAnu shared another polymorphism. Strain Fhon13 showed 98.8% sequence identity (17 polymorphisms) in the 16 S rRNA gene to *L. kunkeei* YH-15. An rRNA-based maximum-likelihood phylogeny showed that *L. kunkeei* and *L. apinorum* Fhon13 were related to *Lactobacillus ozensis*, *Lactobacillus lindneri*, *Lactobacillus sanfranciscensis*,** and *Lactobacillus fructivorans* (fig. 1*a*). For the purpose of this discussion, we have considered *L. apinorum* Fhon13 to be a member of the *L. kunkeei* species complex.

The proteomes of the 13 *L. kunkeei* strains and *L. sanfranciscensis* were clustered into 1,597 protein families, of which 790 were pan-orthologs. The nucleotide sequence alignments of these genes were concatenated and used to infer a phylogeny. The resulting tree suggested that the *L. kunkeei* isolates belong to four distinct microclusters with 2–3 strains per cluster, which we refer to as the A, B, C, and D groups (fig. 1*b*). Strain LMbo was a sister taxon to the A group and LAdo diverged immediately prior to the separation of the A–D groups. As in the 16 S rRNA tree, *L. apinorum* Fhon13 was more divergent and branched off prior to all *L. kunkeei* strains. A comparison of the synonymous substitution frequencies (d*S*) confirmed these relationships, revealing an intragroup divergence of 0.027, 0.031, 0.080, and 0.060 substitutions per site for the A, B, C, and D groups, respectively, as compared with an intergroup divergence of 0.155 (range 0.131–0.197) (supplementary fig. S4, Supplementary Material online). Strain LAdo was the most divergent strain, with a median d*S* value of 0.331 substitutions per site (range 0.301–0.381) compared with the other strains, consistent with its earlier divergence.

### Genome Content and Architecture

#### Massive Genome Reduction

**Fig. 2.—:**
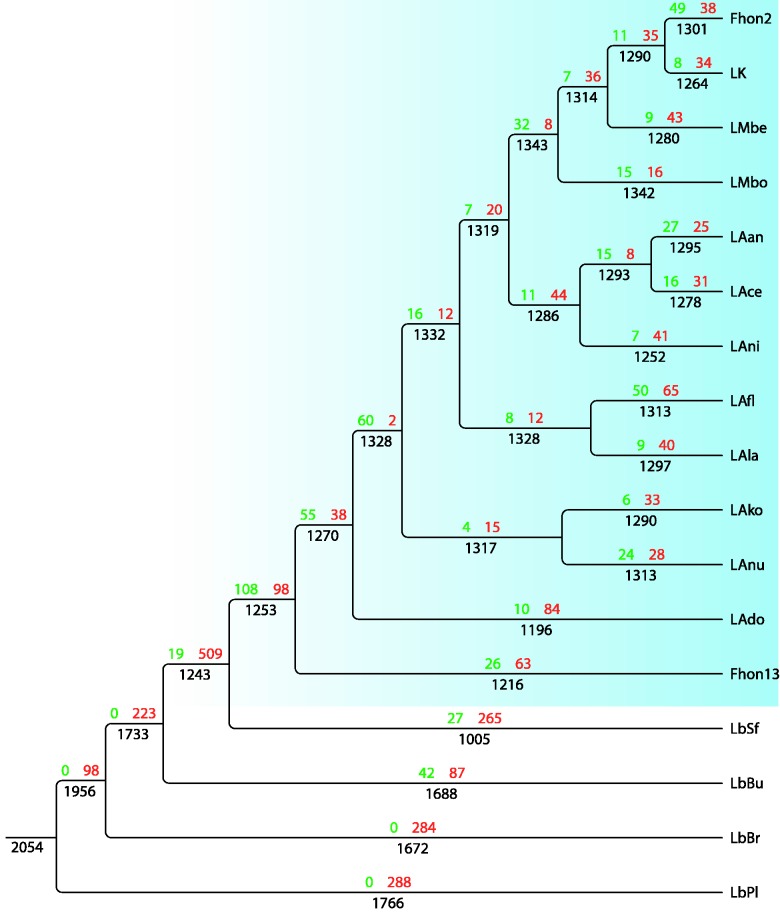
Flux of protein families in *L. kunkeei* and related species. The flux of protein families has been mapped onto the species phylogeny shown in supplementary figure S5, Supplementary Material online. The number of gains of protein families is shown to the left and losses to the right above each branch. The total number of protein families is indicated below each branch. The numbers at the terminal branches include singletons. *Lactobacillus sanfranciscensis* (LbSf), *L. brevis* (LbBr), *L. buchneri* (LbBu) and *L. plantarum* (LbPl). Abbreviations of strain names are defined in table 1.

To learn more about the evolutionary history of *L. kunkeei*, we performed an ancestral reconstruction, that is, an inference of the most likely branches at which protein families have been lost and gained. To this end, we first clustered the proteomes of all species within the Lactobacillaceae and Leuconostocaceae and three additional outgroups into 2,397 protein families. Based on these, we inferred a phylogeny for the *L. kunkeei* species complex and its closest relatives (supplementary fig. S5, Supplementary Material online), onto which we mapped the gains and losses of protein families using generalized parsimony with a penalty for gains of two times the penalty for losses (fig. 2). This analysis allows independent gains of protein families at unrelated nodes or branches in the tree by horizontal gene transfer. Protein families may also be lost and regained.

The inference indicated the loss of 509 protein families in the common ancestor of *L. kunkeei* and *L. sanfrancisensis*, confirming that this clade has evolved by extreme reduction in its genetic repertoire. This may not be surprising since these two species have genome sizes of 1.5–1.8 Mb, as compared with genome sizes of 2.3–3.3 Mb for the outgroup taxa. By normalizing for branch lengths, we found the losses to be 2.6- to 16-fold higher than on the other ancestral branches. Of the 268 lost protein families with assigned functions, 22% have affected the amino acid metabolism, particularly amino acid biosynthesis, and 15% carbohydrate metabolism and transport, consistent with a shift to a nutritionally rich growth habitat. Moreover, all genes for subunits of the pyruvate dehydrogenase complex were lost at this node, suggesting that pyruvate generated from the breakdown of glucose is not channeled further to the TCA cycle, but metabolized to lactate in the fermentation process. The inference further suggested a gain of 19 protein families at this node. Thus, the dramatic losses in the common ancestor of *L. sanfrancisensis* and *L. kunkeei* have not been countered by a corresponding gain, resulting in a large net efflux of genes.

To search for the acquisition of putative host-adaptive traits in the common ancestor of the *L. kunkeei* species complex, we specifically examined the functions of genes gained at the node that separates the *L. kunkeei* species complex from *L. sanfranciscensis.* We inferred a gain of 108 protein families at this node, of which 39 carried assigned functions, including genes for lysozymes and ABC transport systems for ions and oligopeptides (supplementary table S4, Supplementary Material online). About 50 of the protein families acquired at this node have since been maintained in all strains and are thus likely to have been important for the change of lifestyle. These include a gene for beta-fructosidase, which hydrolyzes fructan to fructose, indicating an environmental change in the composition of carbohydrates. The gains were balanced by the loss of 98 families, resulting in no net change in gene number. Of these, 69 carried assigned functions, including genes for cytochrome *bd*-type quinol oxidase, subunits 1 and 2 (supplementary table S5, Supplementary Material online).

Functional differences between the *L. kunkeei* strains were observed for amino acid biosynthetic functions. For example, gene clusters for the biosynthesis of proline, tryptophan, leucine, and arginine showed a scattered distribution pattern (supplementary fig. S6, Supplementary Material online) that did not correlate with the phylogeny of the strains. Additionally, the gene clusters for purine and pyrimidine biosynthesis have been lost in strain LAdo. The identified biosynthetic gene clusters were located in the same genomic regions in all strains, indicative of independent losses.

#### The Genome Architecture Is Functionally Biased

**Fig. 3.—:**
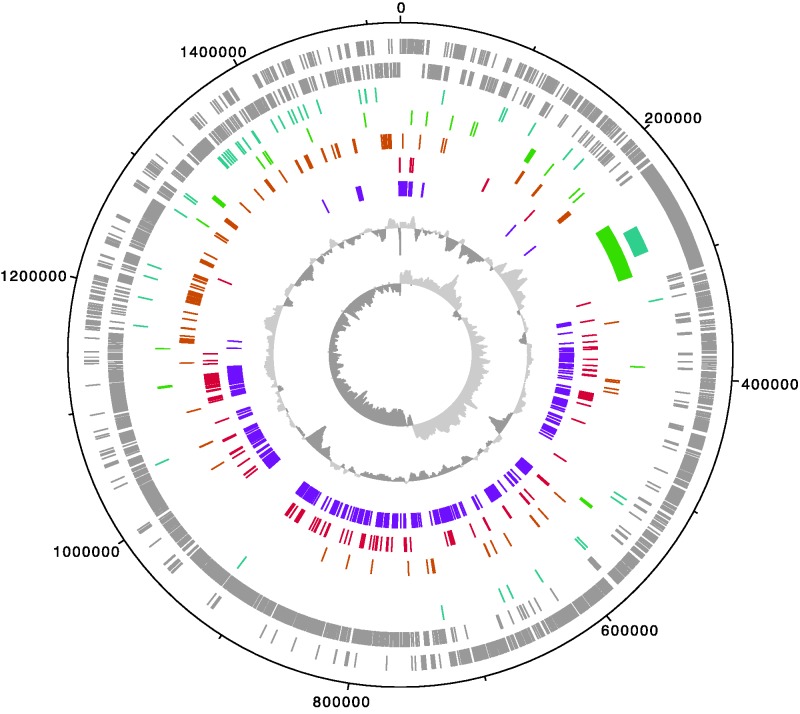
Circular representation of the *L. kunkeei* Fhon2 genome. The color-coding from outer to inner circles represents genes in the forward and reverse strands (gray), genes present in all studied *L. kunkeei* but absent in all other surveyed lactobacilli (blue), genes coding for secreted proteins (green) (as found in Butler et al. [2013]), genes coding for proteins involved in amino acid metabolism (orange) and translation (red) (according to the COG database), genes found in a single copy in all surveyed lactobacilli (purple), GC content and GC skew (gray). Additional *L. kunkeei* genomes are shown in supplementary figure S3, Supplementary Material online.

A visual inspection of the genome architecture in strain Fhon2 indicated that there was an exceptional bias in the functional organization of genes, such that conserved single-copy orthologs present in all of the currently sequenced *Lactobacillus* genomes were mainly situated in the chromosomal half that is flanking *ter* (fig. 3). In contrast, genes for metabolic processes tended to be located in the genomic half that flanks the origin of replication (fig. 3). The genes highly represented in this region code for proteins related to the metabolism of amino acids, such as ABC transporters, permeases, peptidases, and proteases.

**Fig. 4.—:**
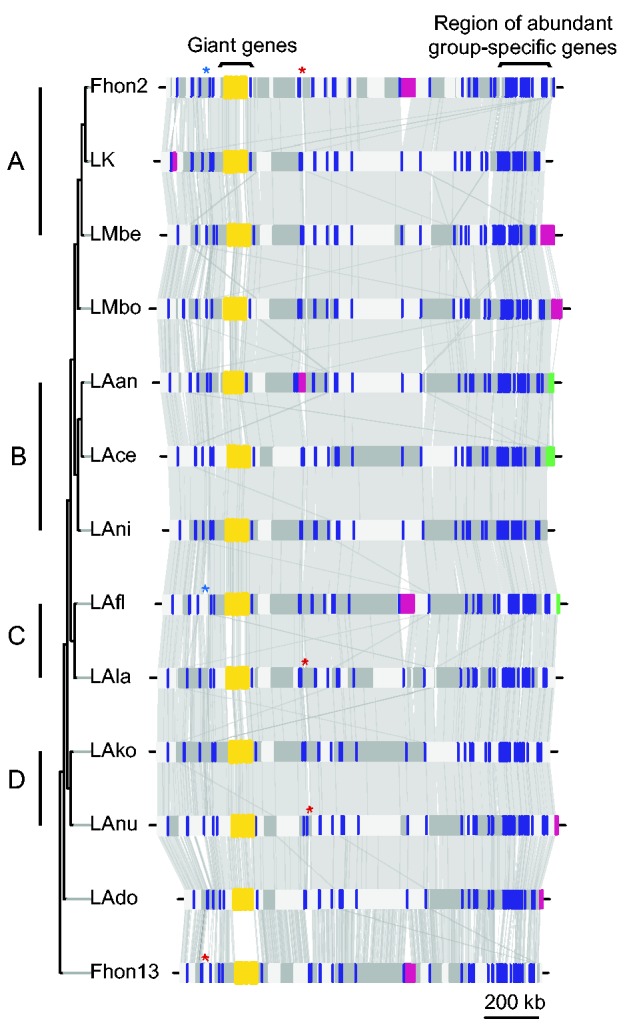
Comparative alignments of the *L. kunkeei* genomes. The color bars and boxes represent phages (pink); plasmids (green); genes identified to be present in all *L. kunkeei* genomes, but no other lactobacilli for which complete genome data are present (blue); and the contiguous giant genes (yellow), which are also unique for these genomes. Asterisks represent CRISPR-*cas* loci, with colors representing different CRISPR types. The alternating horizontal grayscale boxes represent putatively adjacent contigs. Gray links between genomes represent BLASTn nucleotide similarity of at least 80% identity for alignments longer than 300 bases. The tree topology and the microclusters (letters A–D) are as in figure 1*b*.

A similar bias was also observed for the recently acquired and variably present genes located near *ori* versus the conserved ancestral genes located near *ter.* Thus, 40 of the 50 genes present in all strains of *L. kunkeei* but in no other *Lactobacillus* genomes were flanking *ori*, of which 20 were clustered in a 100-kb segment (fig. 4). These genes were often of short, but similar sizes and showed atypically high sequence divergence levels between *L. apinorum* Fhon13 and the *L. kunkeei* strains.

The functionally biased location of genes observed in the Fhon2 genome is a general characteristic of all genomes in the *L. kunkeei* species complex (supplementary fig. S7*a* and *b*, Supplementary Material online). We noted a similar bias in the most closely related strain *L. sanfrancisensis* (supplementary fig. S7*c*, Supplementary Material online), but not in more distantly related species, such as *Leuconostoc mesenteroides* (supplementary fig. S7*d*, Supplementary Material online). The distance to the origin of replication of the genes for amino acid metabolism was significantly different from the distance to *ori* of genes for translation and replication functions (Mann–Whitney tests, *P* < 0.001) in all members of the *L. kunkeei* species complex (supplementary fig. S8, Supplementary Material online).

#### Secreted and Outer Surface Proteins

**Fig. 5.—:**
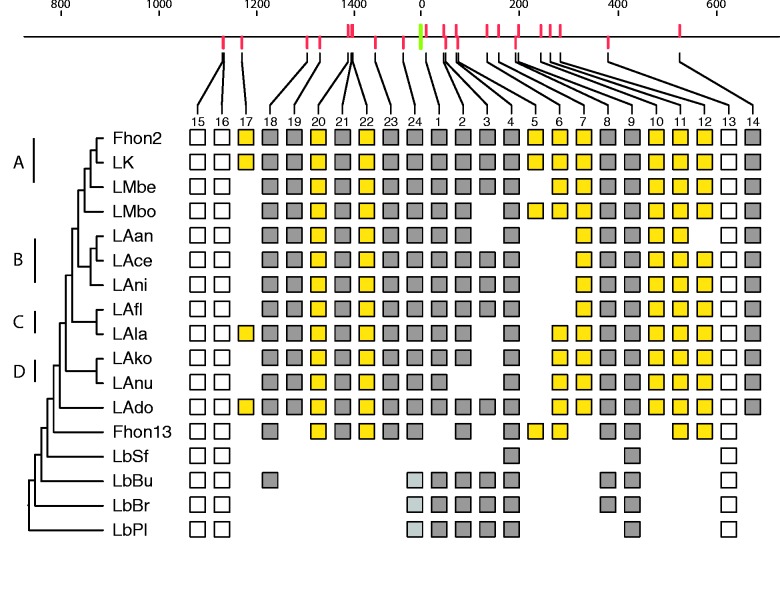
Overview of the location and presence profile of genes coding for secreted proteins. The upper line represents the segment of the Fhon2 genome surrounding the origin of replication (green bar) and the location of the genes coding for secreted proteins (red bars). Tick marks represent the distance in kb. For each gene, its presence/absence profile is shown. Yellow boxes represent genes for secreted proteins that are present in *L. kunkeei* but in no other lactobacilli for which complete genome data are present, gray boxes represent genes with orthologs in other lactobacilli and white boxes represent genes putatively falsely predicted to be secreted. The tree topology and the microclusters (letters A–D) are as in figure 1*b*.

Previous studies have shown that the addition of lipopolysaccharides from *Pseudomonas aeruginosa* to cell cultures of Fhon2 resulted in the production and secretion of 24 extracellular proteins (Butler et al. 2013). The hypothesis was that these proteins are involved in the defense of their niche (the honey crop) against other microbes, and that their secretion is triggered by surface molecules of bacteria such as *Pseudomonas* that are commonly present in flowers. Remarkably, 19 of these genes are located within a region of 300 kb on either side of the origin of replication **(**fig. 5). Of the five genes located more distantly from *ori*, two were coding for ribosomal proteins and one for lactate dehydrogenase. The latter three are the only proteins in the whole set that did not contain a signal peptide, and are thus likely to represent false positives. The 19 genes located in the segment flanking *ori* were generally well conserved, and as many as 14 were identified in all strains of *L. kunkeei.* Consistent with their identification in the secreted pool of proteins, all 19 proteins contain a signal peptide (supplementary table S6, Supplementary Material online). Eight of these secreted proteins have homologs in one or more of the closest relatives to *L. kunkeei,* indicative of vertical inheritance. These include genes for a serine peptidase, a transpeptidase involved in the crosslinking of the Brauns peptide to the peptidoglycan and endopeptidases putatively involved in the hydrolysis of the peptide stem of the peptidoglycan. The identification of secreted proteins involved in the modification of the peptidoglycan is consistent with the hypothesis that these proteins change the surface structure of *L. kunkeei* and/or of other bacteria.

Outer surface proteins that are uniquely present in *L. kunkeei* but not in any of the closely related *Lactobacillus* species are prime candidates for host-adaptive traits. These include two genes for extracellular glucosyltransferase enzymes of more than 1,000 amino acids (nos. 14 and 19), putatively involved in the biosynthesis of α-glucans from sucrose. One of the secreted proteins (no. 23) was present in all strains of *L. kunkeei* but contained no orthologs in any of the previously sequenced *Lactobacillus* genomes. Instead it showed sequence similarity to glycosidases (glycosyl hydrolases which catalyzes the cleavage of the carbohydrate chain of the peptidoglycan) in *Staphylococcus* (BLASTp, *E* < 1 e-52), indicative of horizontal gene transfers. Finally, nine of the secreted proteins were present in all strains of *L. kunkeei* but had no hits to sequences in the public databases.

#### Large, Novel Extracellular Proteins

**Fig. 6.—:**
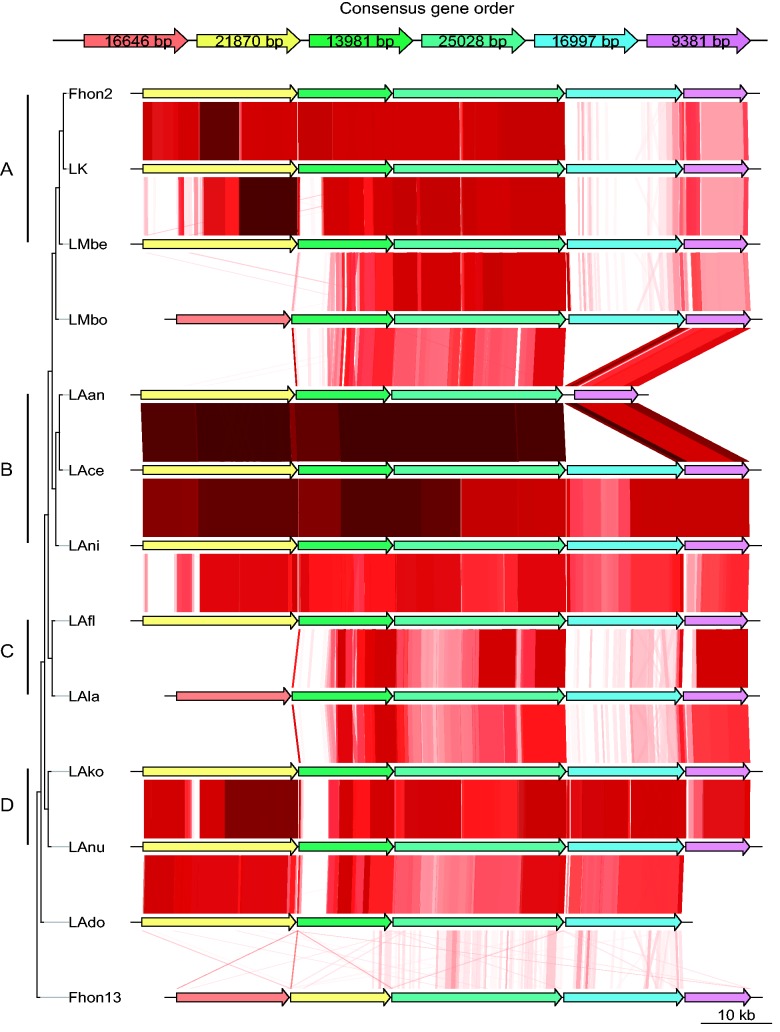
Gene order structures of the segment coding for giant extracellular proteins. Each arrow represents a gene inferred to code for a giant protein, with colors representing distinct protein orthologs. Red lines are shown for tBLASTx hits with more than 50% identity over more than 100 amino acids. White spaces represent either absence of BLAST hits or hits below the established thresholds. Shown at the top is the consensus order of genes and their average lengths. The tree topology and the microclusters (letters A–D) are as in figure 1*b*.

The most remarkable of these putative outer surface and/or secreted proteins of unknown functions belong to a family of proteins that are huge in size, ranging from 3,000 to 9,000 amino acids, and solely present in the *L. kunkeei* species complex. The genes are organized in an array of 4–5 genes and cover a total of 100 kb located about 250 kb downstream of the origin of replication (fig. 6). This region displays atypically high GC-content values and deviating GC-skew values compared with the neighboring segments (fig. 3 and supplementary fig. S3, Supplementary Material online), indicative of acquisition by horizontal gene transfer or strong compositional selection. The encoded proteins showed no similarity to sequences in the Uniprot or NR databases (BLASTp, *E* < 1e-05), and contained no recognizable domains according to the SCOP superfamily classification system. Only the signal peptide at the N-terminal segment, plus a conserved sequence of 60 residues at the C-terminus of unknown function could be detected in all proteins. Psi-BLAST hits were obtained against matrix-binding proteins such as the large EbhA from *Staphylococcus aureus.* Additionally, homology-based structural analysis also detected regions of similarity to EbhA, as well as to streptococcal adhesins, immunoglobulin/albumin-binding domain-like, and catenin alpha-1 (supplementary table S7, Supplementary Material online), suggesting a role in attachment.

The colocation and conserved termini point to a common origin for the entire set of genes, followed by expansion through gene duplication. Notably, the genes in the tandem array were more similar to their positional homologs in the other strains than to the colocated gene copies within each genome, indicating that the duplications occurred prior to the divergence of the strains. In total, six distinct putative protein orthologs could be identified for the colocated genes for the giant proteins, as represented by different colors in figure 6. The genes at the third place in the array (fourth in “consensus gene order,” fig. 6) were the longest and conserved in all strains, with an average gene length of 25 kb. Genes coding for proteins of the other five protein families were missing in one or more strains.

Although there were some indications of a diversification pattern that matched the microcluster relationships, as inferred from the core genome phylogeny, the software Phipack gave positive results for recombination in all ortholog groups, Evidence for recombination is also visualized as patches of different sequence similarity patterns within the genes (fig. 6). For example, the first gene in the array showed strikingly different patterns of similarity within the A phylogroup for the N-terminal, the central, and the C-terminal segment of the gene. Five of these genes were also identified in Fhon13, but these genes were very divergent in sequence and we detected no recombination events between Fhon2 and Fhon13.

### Population Dynamics

#### The *L. kunkeei* and the Host Species Phylogenies Are Incongruent

**Fig. 7.—:**
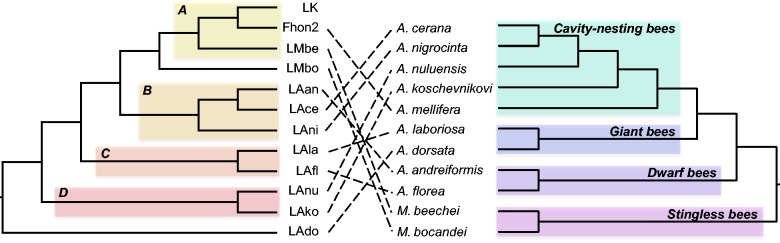
Tanglegram comparing the tree topologies of the *L. kunkeei* strains and their hosts. The tree topology and the microclusters (letters A–D) in the bacterial tree (left) correspond to the maximum-likelihood tree of the concatenated nucleotide alignment presented in figure 1*b.* The host phylogeny (right) has been taken from Arias and Sheppard (2005), Raffiudin and Crozier (2007), and Lo et al. (2010).

To determine whether *L. kunkeei* has codiversified with the bees, we compared the tree topologies of bacteria and bees. On the host side, it has been suggested that dwarf bees, giant bees, cavity nesting bees, and stingless bees belong to four different monophyletic groups (Arias and Sheppard 2005; Raffiudin and Crozier 2007; Lo et al. 2010). Our comparison of the diversification patterns revealed no congruence between bacterial and host tree topologies (fig. 7), suggesting that the *L. kunkeei* strains have not codiversified with their hosts. Nor did we find any correlation between strain relationships and country of isolation.

#### Coinfections Indicate Lack of Host-Specificity

**Fig. 8.—:**
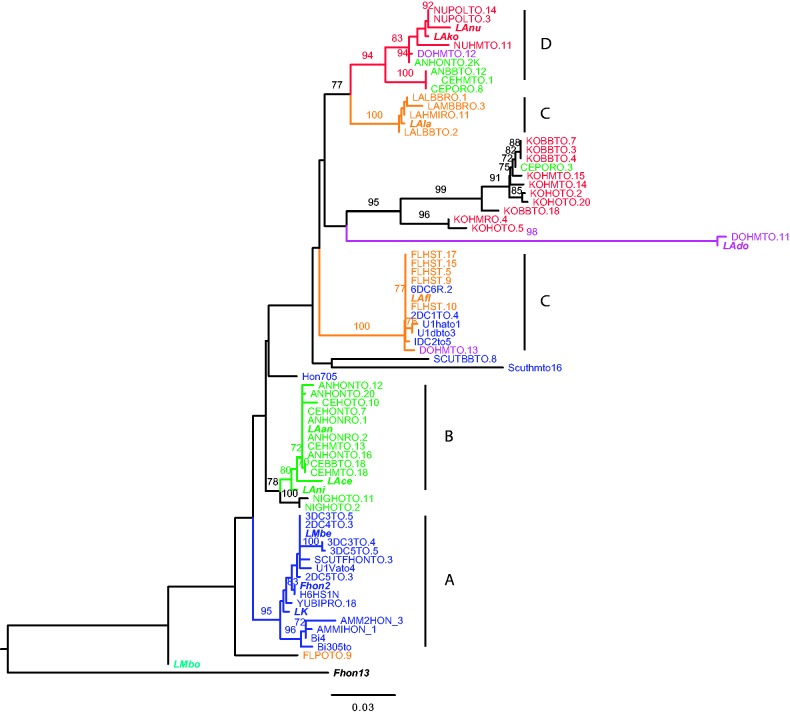
Coinfections inferred from PCR screening of multiple isolates. The phylogeny was inferred from a fragment of the *recG* gene for multiple *L. kunkeei* strains. The colors in the external nodes represent sequences obtained from hosts that belong to the described microclusters: A (blue), B (green), C (orange), D (red), and LAdo (purple). The same colors in the branches represent the microcluster to which the last well-supported ancestor of each of the sequences coming from the genomic data belongs, and all its descendants. The phylogeny was inferred with the maximum-likelihood method.

Given the lack of codiversification between bacterial strains and hosts, we speculated that strains of different genotypes might be present in each host species. If so, there was a risk that we had sampled only a subset of the available strains as genome sequence data were obtained from only one isolate per host. To test for such potentially missed diversity, we amplified with PCR variable segments of the *lepA* and *recG* genes from 27 additional isolates from managed and wild subspecies of *A. mellifera* in Sweden, Kenya and United States, and from 60 isolates from the other 9 host species (supplementary table S8, Supplementary Material online). Overall, we found that most sequences from each host were of the same genotype as those of the sequenced genomes (fig. 8 and supplementary fig. S9, Supplementary Material online), suggesting that the strains selected for sequencing represent the most abundant strains in each host species sample. In cases where the sampling had been done from multiple sites for a given host species (honey, beebread, and/or the honey crop), the same genotype was normally recovered.

As the phylogenies that included the new sequences were inferred from fragments of single genes, we examined only the most highly supported incongruences in detail. For example, most sequences derived from *A. mellifera* clustered with the A-group strain Fhon2, but several sequences also clustered with the C-group strain LAfl. Likewise, most bacterial sequences from *Apis florea* clustered with LAfl, although a few were most similar to LAdo. Finally, most sequences from *Apis cerana* and *Apis andreniformis* clustered with the B-group strains LAan, LAce, and LAni, but a few also clustered with the D-group strains LAko and LAnu. As the same relationships were observed for independently amplified fragments of the *recG* and *lepA* genes, we attribute the presence of mixed genotypes to coinfections rather than to recombination events.

#### Horizontal Transfers between Genotypes Coinfecting the Same Hosts

Next, we tested whether *L. kunkeei* genotypes that can coinfect the same host share a mobile gene pool. Indeed, a prophage of more than 40 kb was identified at the same genomic location in Fhon2, Fhon13, and LAfl (fig. 4, color purple), all of which represent genotypes that we identified in *A. mellifera,* based on our PCR screening of strain diversity. Homologous phage sequences of 40 kb were also identified in the genomes of LMbe and LMbo, both of which infect stingless bee species.

We hypothesized that genotypes occasionally identified in the same host and exposed to the same phage gene pool might also have evolved similar phage defense mechanisms. CRISPR gene cassettes were identified in five strains and could be classified as two different types, according to their gene content and a phylogeny of the *cas1* genes (fig. S10, Supplementary Material online), both of which were present in strain Fhon2. CRISPR gene cassettes of type I–E were identified in strains Fhon2 and LAfl at the same genomic location (fig. 4). CRISPR type II-A was identified in strains Fhon2, LAnu and LAla at the same genomic location, and at a different site in Fhon13. Thus, strain Fhon2 shares similar CRISPR types with strains LAfl and Fhon13, all of which represent genotypes isolated from *A. mellifera.* In contrast, CRISPRs were not shared between strains of the same microclusters.

Interestingly, an analysis of all CRISPR spacers identified in this study found hits for the spacers from strain Fhon2, Fhon13, LAfl, and LAla against the putative phage sequences of strains LMbe and LMbo (supplementary fig. S11, Supplementary Material online). In conclusion, all three strains identified in *A. mellifera* have evolved resistance mechanisms to the same phage present in LMbe and LMbo. The identification of similar prophages and CRISPR elements in bacterial genomes that represent the A-, C-, and Fhon13 genotypes suggests that their co-occurrence in *A. mellifera* is not coincidental.

#### Recombination between Genotypes that Belong to the Same Microcluster

**Fig. 9.—:**
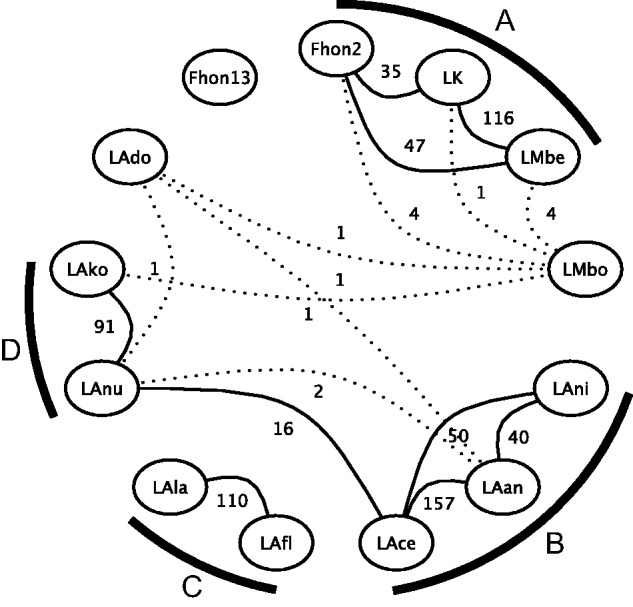
Comparison of single gene tree topologies. The graph depicts supported relationships (>95% bootstrap support) for each pair of taxa in 790 single-copy ortholog phylogenies. Strains that clustered together as sister taxa are shown with connecting lines, where the numbers indicate the total number of trees supporting the clustering. Dotted lines represent arrangements that occurred in less than 1% of the analyzed trees (i.e., 7 or lower). Letters A–D indicate the microcluster affiliation of the strains (as in fig. 1*b*).

To determine whether the strains that are able to coinfect *A. mellifera* also recombine more frequently than other strains in their core genome, which would be indicative of long-term coexistence, we compared the topologies of all 790 single gene trees (fig. 9). None of the 790 trees supported a sister relationship for strains Fhon2 and Fhon13, nor of any of these with strain LAfl. Overall, we observed a strong coherence between the single gene and the concatenated tree topologies regarding the diversification pattern between the microclusters. For example, of the 60 trees inferred from genes longer than 2 kb, 53 supported the monophyly of the four described microclusters with high bootstrap support (>95%). Overall, only 31 of the 790 trees provided strong support (>95%) for a clustering of strains of different microcluster types. The 31 trees with a deviant topology included 9 trees in which LMbo clustered with the A-group strains (as shown for genes longer than 2 kb in supplementary fig. S12, Supplementary Material online), and 18 trees in which LAnu clustered with the B-group strains (as shown for genes longer than 2 kb in supplementary fig. S13, Supplementary Material online).

In contrast, the diversification patterns between strains of the same microcluster were highly variable. For example, the three possible diversification patterns in the A-group were observed in 35–116 single gene trees (bootstrap support values > 95%) (fig. 9). Likewise, all pairwise clusterings of the three strains in the B-group were observed in 40–157 single gene trees (bootstrap support > 95%). The different gene tree topologies did not correlate with either functional categories or overall sequence divergence levels (supplementary fig. S14, Supplementary Material online). For the majority of the single gene trees, in the range of 250 to 500 trees, all three topologies were acceptable according to the AU (Approximately Unbiased), KH (Kishino and Hasegawa), ELW (Expected-Likelihood Weights), and SH (Shimodaira and Hasegawa) tests. For the few trees for which only one topology was included in the confidence set, the tests did not uniformly agree on which one was the most commonly accepted (for full details of the tests results, see supplementary table S9, Supplementary Material online). Unresolved polytomies in tree topologies could be due to multiple, different short recombination tracts within genes, to homogenization by recombination across the entire length of the gene for all three strains, or to scarcity of variation due to strong selective constraints on synonymous sites.

Moreover, the overall ratio at which recombination versus single nucleotide mutations (*r/m)* contribute to the sequence divergences was estimated to 0.376 (95% credibility region: 0.303, 0.514) for all *L. kunkeei* strains (not including Fhon13), when the software ClonalFrame was applied to a set of 25 housekeeping genes. This is in the average range of *r/m* ratios for bacteria (Vos and Didelot 2009) and indicates that nucleotide mutations play a major role in generating sequence divergence for the clade as a whole. Similarly, the software Phipack gave positive results for recombination on 47 of the 1,053 single-copy orthologs in *L. kunkeei* not including Fhon13, that is, for only 4.5% of all genes. We conclude that recombination frequencies are fairly low overall, but high for strains that belong to the same microcluster, irrespectively of the host of isolation.

## Discussion

This study reports the first large-scale, comparative genome analysis of strains belonging to the *L. kunkeei* species complex, which have been isolated from bees and their food products. The genomes are 1.5 Mb in size, which is in the lower bacterial genome size range. Three important features distinguish the *L. kunkeei* strains from other bacteria with similarly sized genomes: 1) Multiple rRNA operons, 2) functionally structured chromosomes, and 3) near identity of the rRNA gene sequences between strains, despite high sequence divergence of protein coding genes, novel gene acquisitions, and gene content variation. Below, we discuss the possible selective forces and mutational mechanisms that may have generated these atypical features.

### Small Genomes with Multiple rRNA Operons

The first striking feature is the presence of multiple rRNA operons in *L. kunkeei* genomes. The sister species, *L. sanfrancisensis*, contains as many as seven rRNA operons in an equally small genome, and this represents the highest known density of rRNA operons in any bacterial genome (Vogel et al. 2011). Although there is no strict correlation between rRNA operon numbers and bacterial genome size, other genomes in this size range typically contain fewer copies of the rRNA gene operons (Vogel et al. 2011). In *Escherichia coli*, the use of multiple rRNA operons has been shown to facilitate shifts from poor to rich growth conditions (Condon et al. 1995). Thus, *L. kunkeei* and its close relatives may have evolved under selection for rapid growth following shifts from poor to nutritionally rich environments. Indeed, *L. kunkeei* grows well in the laboratory with a doubling time of about 55 min, similar to the growth rate of *L. sanfrancisensis* (Ganzle et al. 1998).

As evidenced by *E. coli* and other fast-growing bacteria with large genomes, there is no general, inverse correlation between growth rate and genome size in bacteria (Couturier and Rocha 2006), and therefore we do not believe that the genome size reduction is the result of selection for high growth rates per se. Reductive genome evolution was first described for obligate host-associated pathogens and symbionts, where gene loss and degradation correlate with the shift to the intracellular lifestyles (Andersson JO and Andersson SG 1999; Mira et al. 2001; Toft and Andersson 2010). In contrast, the downsizing of the genomes of free-living, oceanic Alphaproteobacteria and Cyanobacteria has been explained by selection for a low volume-to-surface ratio to increase the concentrations of nutrients inside the cells (Dufresne et al. 2005; Grote et al. 2012). However, neither an intracellular lifestyle nor adaptation to nutrient-poor conditions can account for the genome size reduction in *L. kunkeei.* Rather, we attribute the massive gene loss to specialization to a nutritionally rich diet with a high concentration of simple carbohydrates.

Annual declines in the population size of *L. kunkeei* may also have generated bottlenecks. For example, it is only about 10% of the honeybee colony that hibernates winter and the remaining honeybees in the hive eat from the stored honey very seldom. Furthermore, *L. kunkeei* is unlikely to be able to grow in the absence of fructose, which is available in high amounts from the ingested nectar during the summer months. Consistent with these expectations, we have shown here that the abundance of *L. kunkeei* in the honey crop is drastically reduced during fall and winter. Thus, bottlenecks in the transmission process may be another factor that has contributed to the genome size reduction.

### Functionally Structured Chromosomes

The second remarkable characteristic of the *L. kunkeei* genome is the unique gene organization patterns, in which genes for different functional categories are located in different chromosomal regions. Given the high density of rRNA operons in the *L. kunkeei* genome, we considered the possibility that this architecture reflects gene dosage effects resulting from selection for rapid growth, as observed in the genomes of many other rapidly growing bacteria (Couturier and Rocha 2006). Surprisingly, the functional bias in the *L. kunkeei* genome is just the opposite of the patterns reported previously; it is the metabolic genes rather than genes involved in replication and translation functions that are clustered near the origin of replication. Moreover, we observed no difference in synonymous substitution frequencies for core genes located at different chromosomal positions, as might be expected from variations in gene dosage (Sharp and Li 1987). To our knowledge, such a genomic architecture has not been described previously.

Gene dosage effects are strongest in bacteria with *R* values > 0.5 (*R* = the time it takes to replicate each replichore divided by the minimal doubling time) (Couturier and Rocha 2006). A comparison of the average distance of genes for RNA polymerase, rRNA, and ribosomal proteins divided by half the chromosome size showed no such bias for bacteria with *R* values < 0.25. Based on a growth rate estimate of 55 min and a replication speed of 1,000 nucleotides per second, we calculated an *R* value of 0.23 for *L. kunkeei*, that is, below the value estimated to provide a selective advantage. Moreover, with a genome size of 1.5 Mb and a doubling time of 55 min, only one replication fork is operating during each round of cell division, which reduces the risks for head-on collisions. Altogether, this suggests that the functional bias in gene organization observed here is not caused by selection for an increased copy number of translation genes.

An alternative hypothesis is that multiple rounds of replication initiation generate a higher copy number, and thereby a higher expression level, of genes for transporters and secreted proteins, which might be advantageous during rapid upshifts in growth when new resources become available. A higher copy number of these genes might also increase the likelihood for horizontal gene transfer and recombination. Selection for genes encoding metabolic and transport functions to be located near the origin of replication would indirectly lead to a clustering of the vertically inherited core genes near the terminus of replication.

In addition to the functional bias, we found that most of the “novel genes” in the form of species-specific or group-specific genes in *L. kunkeei* were located in the chromosomal half that flanks the origin of replication, perhaps suggesting that this region is targeted by mobile elements. It has been shown previously that the overall chromosome organization may influence the location of mobile elements (Bobay et al. 2013; Touchon et al. 2014). For example, prophages tend to be located in regions that contain lowly expressed genes as they may be induced to replicate by transcriptional spillover from highly expressed genes. In *E. coli*, the macrodomain located close to the terminus of replication contains many lowly expressed genes and is at the periphery of the nucleoid. As such, it is more accessible to phage integrations (Meile et al. 2011; Tal et al. 2014). In *Bartonella*, a segment encoding a gene transfer agent that is amplified by a phage-derived origin of replication has been identified in the left origin-proximal half of the genome (Berglund et al. 2009; Guy et al. 2013). However, we could not identify either phages or gene transfer agents in the segments flanking the origin of replication. Nor could we identify genes for integrases or a higher abundance of tRNA genes or any other indications of a nonrandom location of integration sites for genomic islands (Williams 2002; Campbell 2003). Thus, although a higher density of horizontally transferred genes in specific regions of the chromosome have also been observed in other bacteria, the mechanisms and selective forces involved in generating the functional bias in genome organization described here are likely to be unique for *L. kunkeei.*

### Near Identity in rRNA Sequences Contrasts with High Sequence Divergence in Protein-Coding Genes

A third characteristic is that the 16 S rRNA sequences are nearly identical between strains, whereas the content and sequences of protein coding genes are highly variable. This feature is shared with phylogenetically unrelated members of the core microbiota of the bee gut (Engel et al. 2014). To explain this paradox it has been proposed that gene transfers occur frequently between all strains in the population, but that only genes that evolve under strong purifying selection, such as the rRNA genes, are similar enough to allow recombination between otherwise divergent groups of bacteria (Engel et al. 2014).

Overall, our study has shown that homologous recombination events occur frequently between closely related strains of the same microcluster, but only rarely between strains of different microcluster affiliations. This could be due to lower frequencies of horizontal gene transfer between strains adapted to different hosts. However, there was no correlation between bacterial and host phylogenies, and we found multiple infections within the same host, which argues against long-term host specialization and isolation. A similar conclusion was obtained from the obligate intracellular and insect-associated *Wolbachia* strains, which recombines freely within supergroups even when sampled from divergent hosts (Klasson et al. 2009), but rarely between supergroups even if the strains infect the same host (Ellegaard et al. 2013). Thus, transmission of bacteria between hosts occurs sufficiently frequently to disrupt patterns of codiversification in both *Wolbachia* and *L. kunkeei* (Werren et al. 1995; Russell et al. 2009).

Despite the lack of codiversification with hosts on an evolutionary time scale, mobile genetic elements have mostly been exchanged by strains isolated from the same host species, irrespectively of microcluster affiliation. Again, a similar observation was made in studies of coinfecting *Wolbachia* strains, which belong to different supergroups, yet share similar bacteriophages (Bordenstein and Wernegreen 2004; Kent et al. 2011). Genes associated with these mobile genetic elements could thus be transferred between divergent strains, facilitating rapid adaptation to local constraints (Boucher et al. 2011). Putative adaptive traits in *L. kunkeei* include the remarkable cluster of giant genes that cover as much as 7% of the genome and other group-specific genes. Elucidating their gene functions and determining why these genes are located in the chromosomal half that flank the origin of replication are interesting avenues for future research.

## Supplementary Material

Supplementary files S1–S3, figures S1–S14, and tables S1–S9 are available at *Genome Biology and Evolution* online (http://www.gbe.oxfordjournals.org/).
